# Mammalian Mucosal α-Glucosidases Coordinate with α-Amylase in the Initial Starch Hydrolysis Stage to Have a Role in Starch Digestion beyond Glucogenesis

**DOI:** 10.1371/journal.pone.0062546

**Published:** 2013-04-25

**Authors:** Sushil Dhital, Amy Hui-Mei Lin, Bruce R. Hamaker, Michael J. Gidley, Anbuhkani Muniandy

**Affiliations:** 1 Whistler Center for Carbohydrate Research, Department of Food Science, Purdue University, West Lafayette, Indiana, United States of America; 2 Centre for Nutrition and Food Sciences and Australian Research Council Centre of Excellence in Plant Cell Walls, The University of Queensland, Brisbane, Queensland, Australia; National Institute of Agronomic Research, France

## Abstract

Starch digestion in the human body is typically viewed in a sequential manner beginning with α-amylase and followed by α-glucosidase to produce glucose. This report indicates that the two enzyme types can act synergistically to digest granular starch structure. The aim of this study was to investigate how the mucosal α-glucosidases act with α-amylase to digest granular starch. Two types of enzyme extracts, pancreatic and intestinal extracts, were applied. The pancreatic extract containing predominantly α-amylase, and intestinal extract containing a combination of α-amylase and mucosal α-glucosidase activities, were applied to three granular maize starches with different amylose contents in an *in vitro* system. Relative glucogenesis, released maltooligosaccharide amounts, and structural changes of degraded residues were examined. Pancreatic extract-treated starches showed a hydrolysis limit over the 12 h incubation period with residues having a higher gelatinization temperature than the native starch. α-Amylase combined with the mucosal α-glucosidases in the intestinal extract showed higher glucogenesis as expected, but also higher maltooligosaccharide amounts indicating an overall greater degree of granular starch breakdown. Starch residues after intestinal extract digestion showed more starch fragmentation, higher gelatinization temperature, higher crystallinity (without any change in polymorph), and an increase of intermediate-sized or small-sized fractions of starch molecules, but did not show preferential hydrolysis of either amylose or amylopectin. Direct digestion of granular starch by mammalian recombinant mucosal α-glucosidases was observed which shows that these enzymes may work either independently or together with α-amylase to digest starch. Thus, mucosal α-glucosidases can have a synergistic effect with α-amylase on granular starch digestion, consistent with a role in overall starch digestion beyond their primary glucogenesis function.

## Introduction

Starch is the major dietary carbohydrate source of glucose for the human, and the rate and extent of starch digestion is associated with glycemia-related problems such as diabetes and other metabolic syndrome conditions. To generate dietary glucose from starchy foods, salivary and pancreatic α-amylase and four small intestine mucosal α-glucosidase subunits are employed in the human body. α-Amylase hydrolyzes starch by an endo mechanism at inner α-1,4 glucosidic linkages and produces linear and branched maltooligosaccharides [1]. The mucosal α-glucosidases are two membrane bounded protein complexes, maltase-glucoamylase (MGAM) and sucrase-isomaltase (SI). All four subunits, N- and C-terminal subunits of MGAM and SI complexes, hydrolyze α-1,4 glucosidic linkages from the non-reducing end of α-amylase-degraded starch molecules and produce free glucose [2]–[4]. In addition to its well-known maltase activity [2], Ct (C-terminal subunit) MGAM was termed glucoamylase due to its activity on longer chain oligomers [5], and Nt (N-terminal subunit) SI was named as isomaltase because of its debranching activity [6].

The conventional view of starch digestion has suggested that α-amylase is the limiting digestive enzyme that determines digestion rate. The role of the mucosal α-glucosidases has been thought to simply and passively convert the post-α-amylase dextrins to glucose. However, we reported that recombinant Nt-MGAM α-glucosidase can digest even granular starch and the four individual α-glucosidases digest gelatinized starch molecules to glucose without the aid of α-amylase, albeit at a slow rate for granular starch [7]–[10]. One subunit, Ct-MGAM, digested starch molecules *in vitro* to nearly 80% [8]. This supports the hypothesis that human mucosal α-glucosidases may act together with α-amylase to digest starch, or at the very least provide an alternative pathway for starch digestion when luminal salivary and pancreatic α-amylase activity is inhibited or reduced because of immaturity and malnutrition [11], [12]. We proposed that human α-amylase is not required for granular starch digestion, but amplifies its digestion by providing favored substrates for mucosal α-glucosidases [10]. However, we did not have evidence that the two enzyme types, α-amylase and the mucosal α-glucosidases, might work together to better digest starch at the initial hydrolysis step.

Our objective here was to distinguish the complimentary digestion roles of α-amylase and mucosal α-glucosidase on granular starch. In this study, we first applied the individual recombinant α-glucosidase subunits, Nt-MGAM, Ct-MGAM, Nt-SI and Ct-Si, to granular normal maize to see whether all four mucosal α-glucosidase subunits can digest granular starch to glucose without the pre-hydrolysis of α-amylase. Then, we chose three maize granular starches with different amylose contents as *in vitro* test substrates. They were hydrolyzed by two types of enzymes extracted from pancreatin powder and intestinal powder, respectively. The pancreatic extract represents predominantly α-amylase, and the intestinal extract represents a mixture of α-amylase [13] and mucosal α-glucosidases. With the objective to understand whether there is a synergistic effect of the two enzyme types on granular starch digestion, we examined α-glucogenesis (glucose production), released maltooligosaccharide amounts, and starch structural changes including granular morphology, molecular weight distribution, and changes in supramolecular structure, as monitored by X-ray diffraction patterns and thermal properties.

## Materials and Methods

### Materials

Normal and waxy maize were obtained from Tate and Lyle (Decatur, IL, U.S.A.) and high-amylose maize was from Ingredion Inc. (Hylon-7®, Bridgewater, NJ, U.S.A.). Normal and high-amylose maize contain about 26 and 70% amylose, respectively; waxy maize is essentially amylose-free. Pancreatin powder (P-7545, from porcine pancreas), intestinal powder (I-1630, from rat intestine), and soluble potato starch (S-2630) were purchased from Sigma Chemical Co. (St. Louis, MO, U.S.A.). Recombinant human Nt-MGAM, Ct-MGAM, and Nt-SI and recombinant mouse Ct-Si were obtained as described previously [8]. The glucose oxidase-peroxidase (GOPOD) assay kit was purchased from Megazyme International Ireland Ltd. (Wicklow, Ireland), the amylase activity assay kit (K711-100) was from BioVision Inc. (Milpitas, CA, U.S.A.), and the Micro BCA™ protein assay kit was from Thermo Scientific (Rockford, IL, U.S.A.).

### Starch Digestion with Individual Recombinant Mucosal α-glucosidases

Normal maize was dispersed in a 10 mmol/L phosphate buffer (pH 7.0, 10 mg of starch dry mass/mL). Aliquots of starch suspension (10 µL) were transferred to micro-centrifuge tubes and incubated with individual mucosal α-glucosidases (200 units) for different time intervals (12, 24, and 48 h) in a shaking water bath set at 37°C and 80 oscillations per minute. The parameters (reaction time periods) were chosen based on the pre-tests (data not shown), in order to generate significant amounts of glucose. α-Glucosidases were inactivated by heating in a boiling water bath for 10 min. The released glucose amount was determined by the glucose oxidase-peroxidase (GOPOD) assay [14]. The digestion experiments were done in triplicate. One unit of activity was defined as the amount of glucose (µg) that is released from 10 µL of 50 mmol/L maltose at 37°C in 5 min. The released glucose amount was determined by the GOPOD assay. Assays were done in triplicate.

### Enzyme Extraction

Pancreatin (10 mg/mL) and intestinal powder (20 mg/mL) were dispersed in a phosphate buffer (10 mmol/L, pH 6.8, 0.02% (w/v) sodium azide). After vortex treatment for 5 min, the mixture was centrifuged at 10,000 *g* for 30 min at 4°C. The clear supernatant was kept at 4°C and subsequently used for the experiment. The pancreatic and intestinal extract contained 5.24 and 8.74 mg/mL protein respectively, as determined by using a Micro BCA™ protein assay kit.

### Enzyme Activity of Pancreatic and Intestinal Extracts

The activity was measured on 1% (w/v) soluble potato starch further solubilized by heating in a boiling water bath for 20 min. Starch solutions (100 µL) were mixed with enzyme extracts (pancreatic or intestinal extracts) and incubated at 37°C for 10 min and then immediately heated in a boiling water bath (10 min) to inactive enzymes. The heated tubes were cooled to room temperature, and then the glucose amount was determined by the GOPOD assay, the reducing sugar amount was determined by the Somogyi-Nelson method [15], [16]. Enzyme activity was expressed as the amount of protein (µg in enzyme solution) required to release 1 µmol of reducing sugar (expressed as glucose) in 10 min from 100 µL of 1% (w/v) soluble potato starch. All the assays were done in triplicate.

### Endo- and exo-hydrolytic Activities of Intestinal Extract

Intestinal extract contains both α-amylase, with endo-hydrolytic activity, and the mucosal α-glucosidases, with exo-hydrolytic activities. To investigate their individual contribution to total hydrolytic activity, the endo- and exo-activities were determined by the BioVision amylase activity assay and maltase activity assay, respectively. The BioVision amylase assay uses ethylidene-pnitrophenol (pNP)-maltoheptaoside as the substrate. Once the substrate is specifically cleaved by α-amylase activity, the smaller fragments produced are acted upon by the α-glucosidases, which causes the release of the chromophore that is then be measured at 405 nm. Exo-hydrolytic activity, presented as maltase activity, was determined by incubating 10 µL extract with 10 µL of 50 mmol/L maltose for 5 min at 37°C and released glucose was measured by the GOPOD assay kit.

### Starch Hydrolysis by Pancreatic and Intestinal Extracts

The granular starches (2 g, dry weight) were incubated with pancreatic or intestinal extract (30 units, in 10 mmol/L phosphate buffer, pH 6.8) in a shaking water bath (37°C, 120 oscillations per minute) for different time periods (2, 4, 8 and 24 h). Due to the higher reaction rate obtained from the pre-tests (data not shown), the reaction time period here was shorter than the hydrolysis time period for individual mucosal α-glucosidases. Immediately after the designated time period, the tubes were centrifuged at 4,500 *g* for 30 min at 4°C. After centrifugation, supernatants were heated in a boiling water bath for 10 min and glucose amount was determined by the GOPOD assay [14], [17], reducing sugar was determined by the Somogyi-Nelson method [15], [16], and maltose, maltotriose and maltotetraose amounts were quantified by using high-performance anion-exchange chromatography (HPAEC). Residues were washed with deionized water for five times and finished with absolute ethanol, and air-dried at 30°C for structural analysis.

The HPAEC (Dionex, Sunnyvale, CA) was coupled with an electrochemical detector (ED40, Dionex, Sunnyvale, CA). The supernatants were filtered through a 0.45 µm pore size filter and were injected onto a CarboPac PA-1 pellicular anion exchange column (Dionex, CA, USA) that was pre-equilibrated in 150 mmol/L NaOH at 1.0 mL/min. Chromatographic separation of the enzyme-treated samples was achieved using a linear gradient of 100% of 150 mmol/L NaOH (eluent A) to 35% of 600 mmol/L sodium acetate in 150 mmol/L NaOH (eluent B) in 35 min. Serial concentrations of maltose, maltotriose, and maltotetraose were applied to make calibration curves of sugar concentration and peak area. Comparison of the amounts of each sugar was analyzed by one-way ANOVA and followed by Tukey’s test at a confidence interval of 95%.

### Morphology of Starch Residues

Starch residues were sprayed on circular metal stubs previously covered with double–sided adhesive and platinum coated at 5 nm thickness under vacuum. Coated samples were observed through scanning electron microscope (JEOL-JSM 6300, JEOL Ltd, Tokyo, Japan) under an accelerating voltage of 5 kV.

### Molecular Size Distributions of Starch Residues

The size distributions of debranched and un-debranched starch molecules in the native and enzyme degraded (2, 4, 8 and 12 h) remnant starches were analyzed using a high performance size-exclusion chromatography (HPSEC) system (Agilent 1100 Series SEC system, Agilent Technologies, Waldbronn, Germany) with refractive index detection (PN3140, PostNova Analytics, Landsberg, Germany) as described previously [18], [19]. Briefly, starch was gelatinized in dimethyl sulfoxide (DMSO) solution containing 0.5% (w/w) lithium bromide (LiBr) at 80°C in a thermomixer (Thermomixer Comfort, Eppendorf, Hamburg, Germany) for 24 h. For debranched molecules, gelatinized starch molecules (4 mg) were precipitated in ethanol, converted to an aqueous system with acetate buffer (pH 3.5) and treated with isoamylase. The debranched molecules were precipitated with 5 mL absolute ethanol, centrifuged (4000 *g*, 10 min), and then dissolved in 1 mL DMSO/LiBr at 80°C in the thermomixer for 3 h for the chromatogram analysis.

For the un-debranched molecules, a set of columns consisting of GRAM precolumn, GRAM 30, and GRAM 3000 (PPS Polymer Standards Service GmbH, Mainz, Germany) was used to elute samples with DMSO with 0.5% w/w LiBr at 0.3 mL/min at 80°C. For the de-branched molecules, a set of columns consisting of GRAM pre-column, GRAM 100, and GRAM 1000 was used to elute samples in the same eluent at 0.6 mL/min at 80°C. The RI detector was set at 45°C. A series of pullulan standards (Polymer Standard Services, Mainz, Germany) with varying molecular weights ranging from 342 to 2,350,000 Da were used for the calibration of molecular size with elution volume. The resulting HPSEC chromatographs were analyzed using PSS WinGPC Unity software (Polymer Standard Services, Mainz, Germany) and normalized to yield the same peak height of the highest peak of the un-debranched and debranched starch distributions.

Size distribution *w* (log *V*_h_) was calculated from the detector (RI) signal and plotted against *R*_h_, hydrodynamic radius (nm) calculated from hydrodynamic volume *V*_h_ with 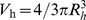 [18], [20]. For the un-debranched molecules, the size range where apparent R_h_ >200 nm is the amylopectin region. For the debranched molecules, the chromatographs were also presented in terms of degree of polymerizations (DP). The amylose branches are in the range of ≈3.5–60 nm (DP≈200–3000); the amylopectin branches are smaller than 3.5 nm. The smaller outer branches such as those confined to one lamaella (A and B1 chains, DP≈10–30) have R_h_ values of about 2 nm, and the longer inner branches that span more than one lamella (B2, B3,…., DP≈30–60) have R_h_ values of about 2.5 nm.

### X-ray Diffraction Pattern of Starch Residues

The X-ray diffraction measurements of samples were made with a Panalytical X’Pert Pro diffractometer (Almelo, Holland). The instrument was equipped with a copper X-ray generator (λ = 1.54 Å), programmable incident beam divergence slit and diffracted beam scatter slit, and an X’celerator high speed detector. X-ray diffraction patterns were acquired at room temperature over the 2θ range of 3° to 38° with a step size of 0.03, and a count time of 400 seconds per step**.** Percentage crystallinity (relative crystallinity) was calculated as the ratio of the total peak area to the total diffraction area [21], [22]. The relative crystallinity contributed by each major peak was calculated using Traces version 3.01 software (Diffraction Technology Pty Ltd., Mitchell, ACT, Australia).

### Thermal Properties of Starch Residues

The gelatinization properties of starch residues were analyzed using a differential scanning calorimeter (DSC 1, Mettler Toledo, Schwerzenbach, Switzerland). Each sample (≈4 mg) was mixed with ≈12 mg of deionized water in an aluminum pan (40 µL), which was then hermetically sealed. The pans were held at 10°C for 5 min and then heated to 120°C at 5°C/min. The onset (T_O_), peak (T_P_), and conclusion temperatures (T_C_) and the enthalpy of gelatinization (ΔH) were determined using the built-in software (STARe System, Mettler Toledo, Schwerzenbach, Switzerland).

## Results

### Starch Digestion with Individual Recombinant Mucosal α-glucosidase Subunits

**Figure 1:**
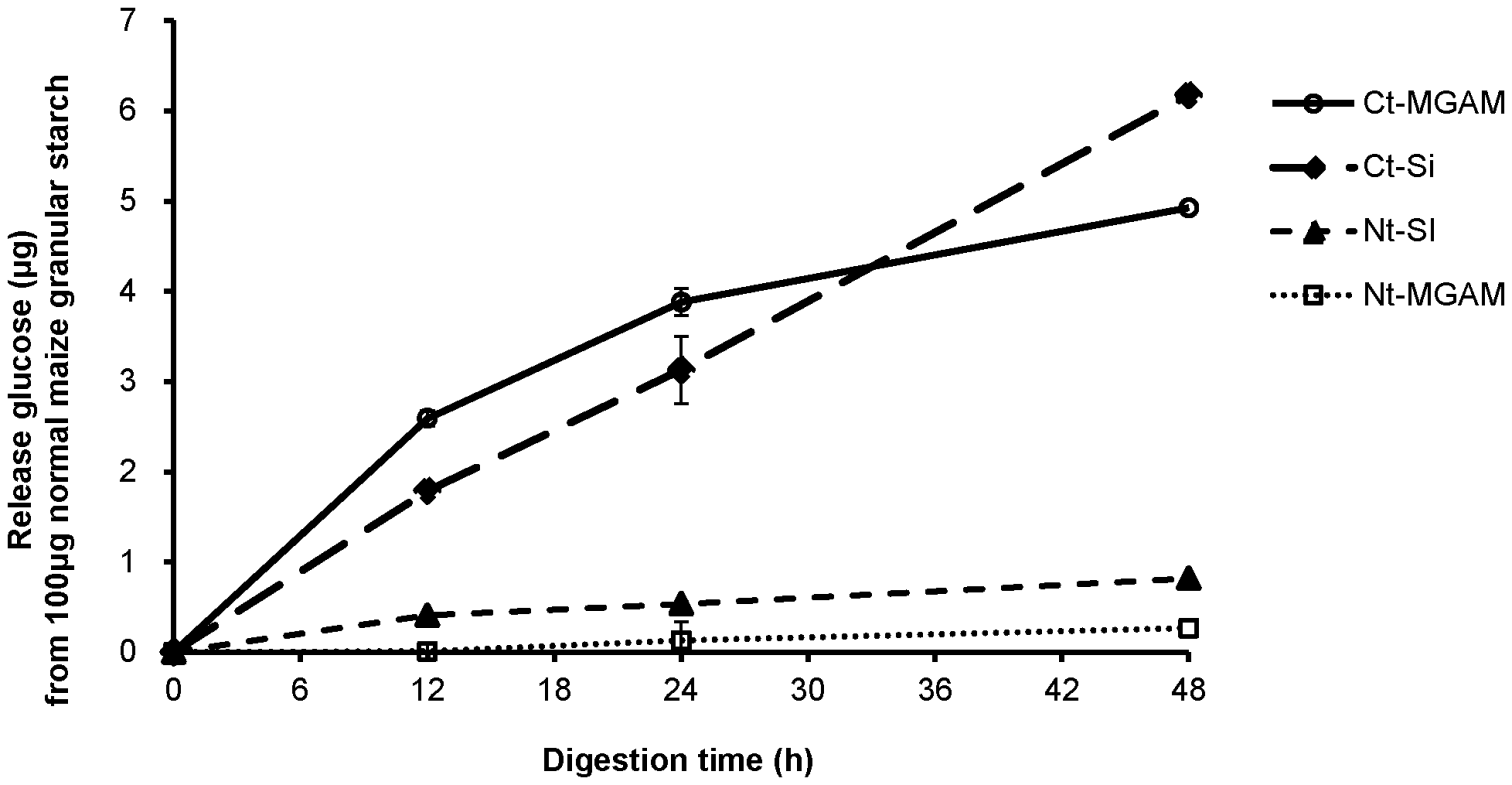
Direct digestion profiles of individual recombinant mucosal α-glucosidase subunits. Granular normal maize was incubated with recombinant mucosal α-glucosidase including human Nt-MGAM, Ct-MGAM, Nt-SI, and mouse Ct-Si at 37°C for 48 h. The released glucose amount was determined by the GOPOD assay. Values are mean ± standard deviation in triplicate analysis.

The production of glucose from normal maize starch granules digested with the four individual recombinant mucosal α-glucosidases, Nt-MGAM, Nt-SI, Ct-MGAM, and Ct-Si, is shown in Fig. 1. Without α-amylase participation, all four individual mucosal α-glucosidase subunits digested granular starch and released some amount of glucose at different rates. The two C-terminal subunits, Ct-MGAM and Ct-Si, were comparably much more active in direct granular starch digestion than the N-terminal subunits (ca 6% conversion to glucose after 48 h compared with <1%).

### Endo- and Exo-hydrolytic Activities of Pancreatic and Intestinal Extracts

Pancreatic extract had a relatively higher endo-hydrolytic activity and a significantly lower exo-hydrolytic activity than intestinal extract. Pancreatic and intestinal extracts produced about 240 and 170 nmol nitrophenol (NP) from ethylidene-pNP-maltoheptaoside per minute, and produced about 50 and 1060 nmol glucose from maltose per minute, respectively (Table 1).

**Table 1:** Endo- and exo-hydrolytic activities of pancreatic and intestinal extracts.

	PancreaticExtract	IntestinalExtract	*P* value^1^
***Endo-hydrolytic activity*** 2			
Nitrophenol (nmol/min)	240.3±7.7^4^	169.7±22.1	0.0509
***Exo-hydrolytic activity*** 3			
Glucose (nmol/min)	51.0±7.4	1060.3±29.3	0.0004

1 The statistical assay was achieved using t-Test at a significance level of 5%.

2 Determined by the BioVision amylase activity assay.

3 Determined by the maltase activity.

4 The values are mean ± standard deviation of triplicated measurements.

### Starch Digestion by Pancreatic and Intestinal Extracts (Glucogenesis)

**Figure 2:**
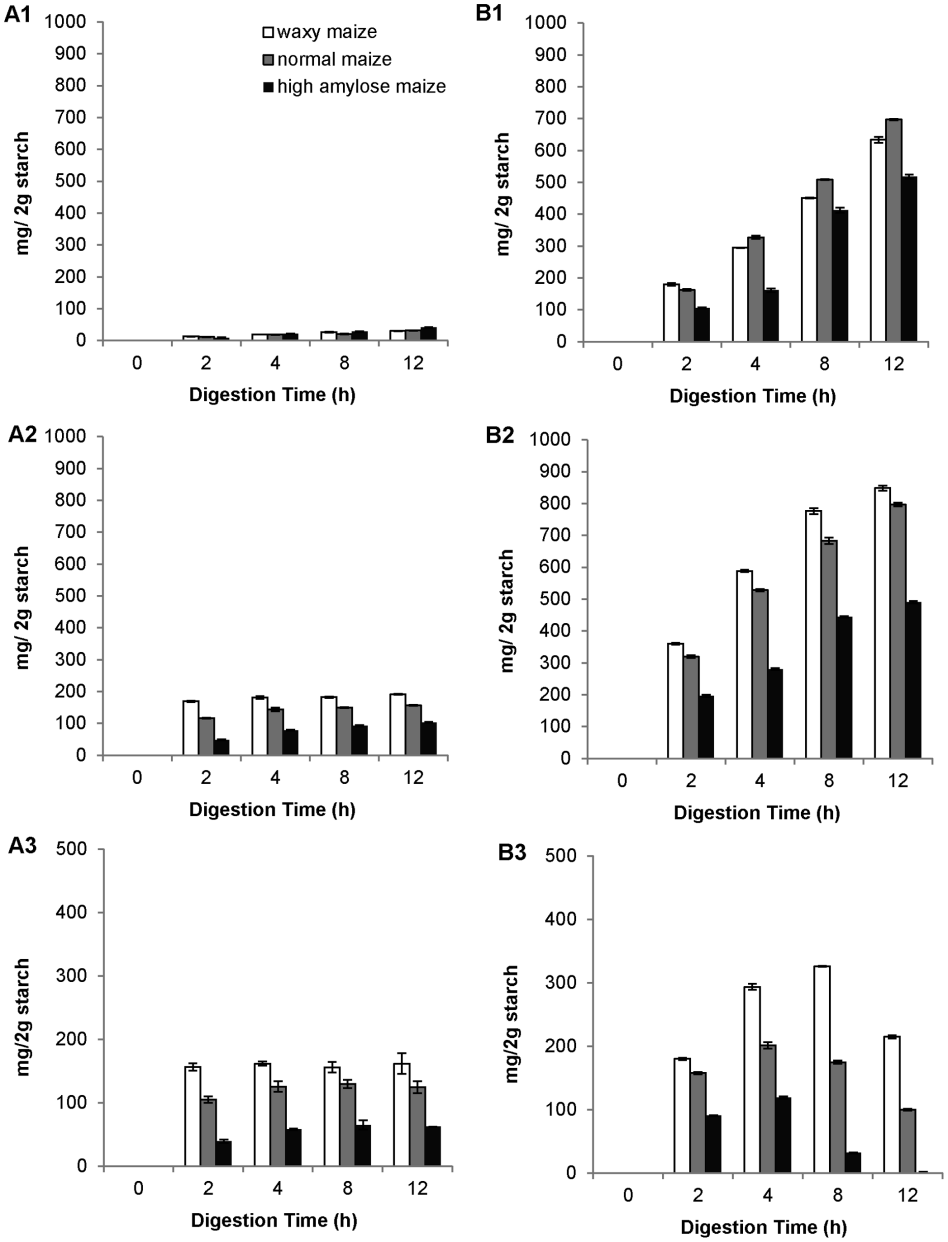
Kinetics of pancreatic and intestinal hydrolysis on three granular maize starches. Waxy (white bar), normal (grey bar), and high-amylose maize (black bar) starches were incubated with pancreatic (A1, A2, A3) and intestinal (B1, B2, B3) extracts respectively for 2, 4, 8, and 12 h at 37°C. The released glucose (A1, B1) and reducing sugar (A2, B2) were determined by GOPOD and Somogyi-Nelson methods, respectively. Maltooligosaccharides (A3, B3) amount was calculated as total reducing sugar minus glucose amount. The values are means ± standard deviation in triplicate analysis.

Both pancreatic and intestinal extracts released glucose from all three granular maize starches (Fig. 2, A1 and B1), although the pancreatic extract produced very little glucose (less than 30 mg per two grams of starch after 12 h). It is consistent with the low level of maltase activity. Intestinal extract produced up to 700 mg glucose from two grams normal maize starch. Glucogenesis by intestinal extract followed the order of normal maize>waxy maize>high-amylose maize (Fig. 2, B1). During the hydrolysis period, the relationship between glucogenesis and reaction time of intestinal extract digestion was nearly linear.

### Reducing Sugar Production

The intestinal extract produced a higher amount (≈500 to 850 mg from 2 g starch) of reducing sugar than the pancreatic extract (≈100 to 200 g from 2 g starch, Fig. 2, A2 and B2) (note that the applied activity was normalized by reaction on soluble starch). The susceptibility to both pancreatic and intestinal extracts was in the same order: waxy maize>normal maize>high-amylose maize. Pancreatic extract hydrolysis reached a plateau after 2–4 h (Fig. 2, A2); an approximate linear relationship was apparent with intestinal extract hydrolysis between reducing sugar amount and reaction time (Fig. 2, B2).

### Maltooligosaccharides Production

The released maltooligosaccharide amount (Fig. 2, A3 and B3) was calculated by subtracting the amount of released glucose (Fig. 2, A1 and B1) from the amount of total reducing sugar (Fig. 2, A2 and B2). The intestinal extract released more maltooligosaccharides than the pancreatic extract from waxy and normal maize starches (Fig. 2, A3 and B3; the statistical analysis results were shown in Table S1). The maltooligosaccharides released by both pancreatic and intestinal extracts were in the same order: waxy maize>normal maize>high-amylose maize (Fig. 2, A3 and B3; the statistical analysis results were shown in Table S2). Pancreatic extract reached a limitation on producing maltooligosaccharides at 2–4 h; while intestinal extract kept producing maltooligosaccharides up to 8 h for waxy maize and normal maize whereas, in high-amylose maize, it dropped at about 4 h.

**Figure 3:**
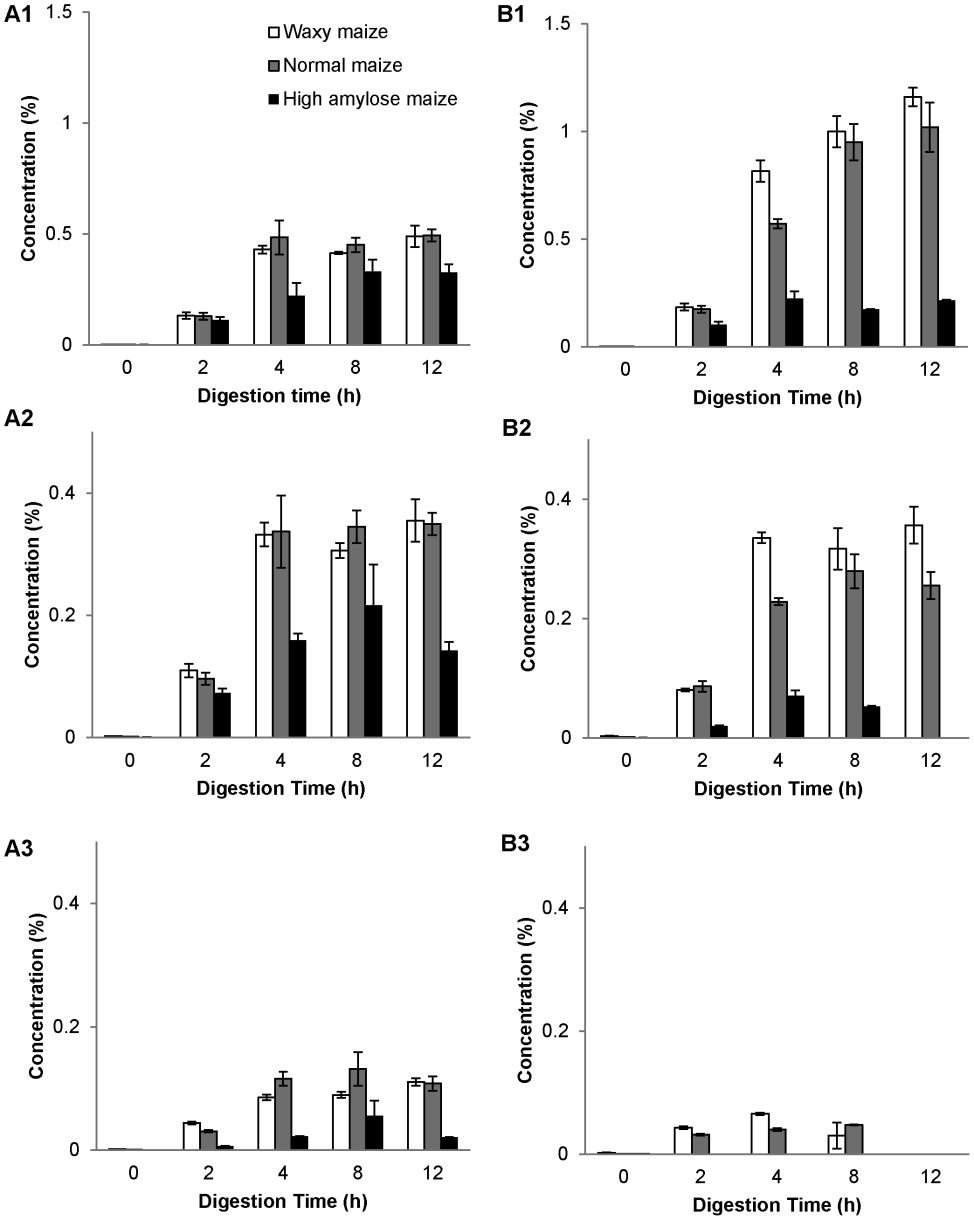
Kinetics of pancreatic and intestinal hydrolysis on releasing maltose, maltotriose and maltotetraose from granular starches. Waxy (white bar), normal (grey bar), and high-amylose maize (black bar) starches were incubated with pancreatic (A1, A2, A3) and intestinal (B1, B2, B3) extracts respectively for 2, 4, 8, and 12 h at 37°C. The released amounts of maltose (A1, B1), maltotriose (A2, B2), and maltotetraose (A3, B3) were determined by HPAEC. The values are means ± standard deviation in duplicated analysis. The statistical analysis results were listed in Table S3.

The released major sugars of maltooligosaccharides including maltose, maltotriose, and maltotetraose were further quantified by HPAEC (Fig. 3). The quantity was in an order of maltose>maltotriose>maltotetraose (The significance of difference was in a confidence interval of 95%) in both extract-treated starch remnants, and the order was the same in all three maize starches as well. Pancreatic treated starches had higher amount of maltotetraose than intestinal extract treated starches (Fig. 3, A3 and B3; *P = *0.004, confidence interval = 95%). There was no significant increase of maltose and maltotriose amount after 4 h for all three maize starches, and high-amylose maize had a drop of maltotriose and maltotetraose production at 12 h (Fig. 3, A1 and A2; statistical analysis results were shown in Table S3). Intestinal extract-treated waxy and normal maize had an increase in maltose production during the 12 h period, but high-amylose maize did not have a significant increase (Fig. 3, B1; Table S3). For maltotriose, intestinal extract-treated waxy maize did not have significant change after 4 h (Table S3), normal maize had a drop in amount at 12 h, and high-amylose maize dropped at 8 h (Fig. 3, B2). Intestinal extract-treated waxy and normal starches had lower maltotetraose amount; digested high-amylose maize remnants did not contain detectable maltotetraose (Fig. 3, B3).

### Morphology of Starch and the Residues

**Figure 4:**
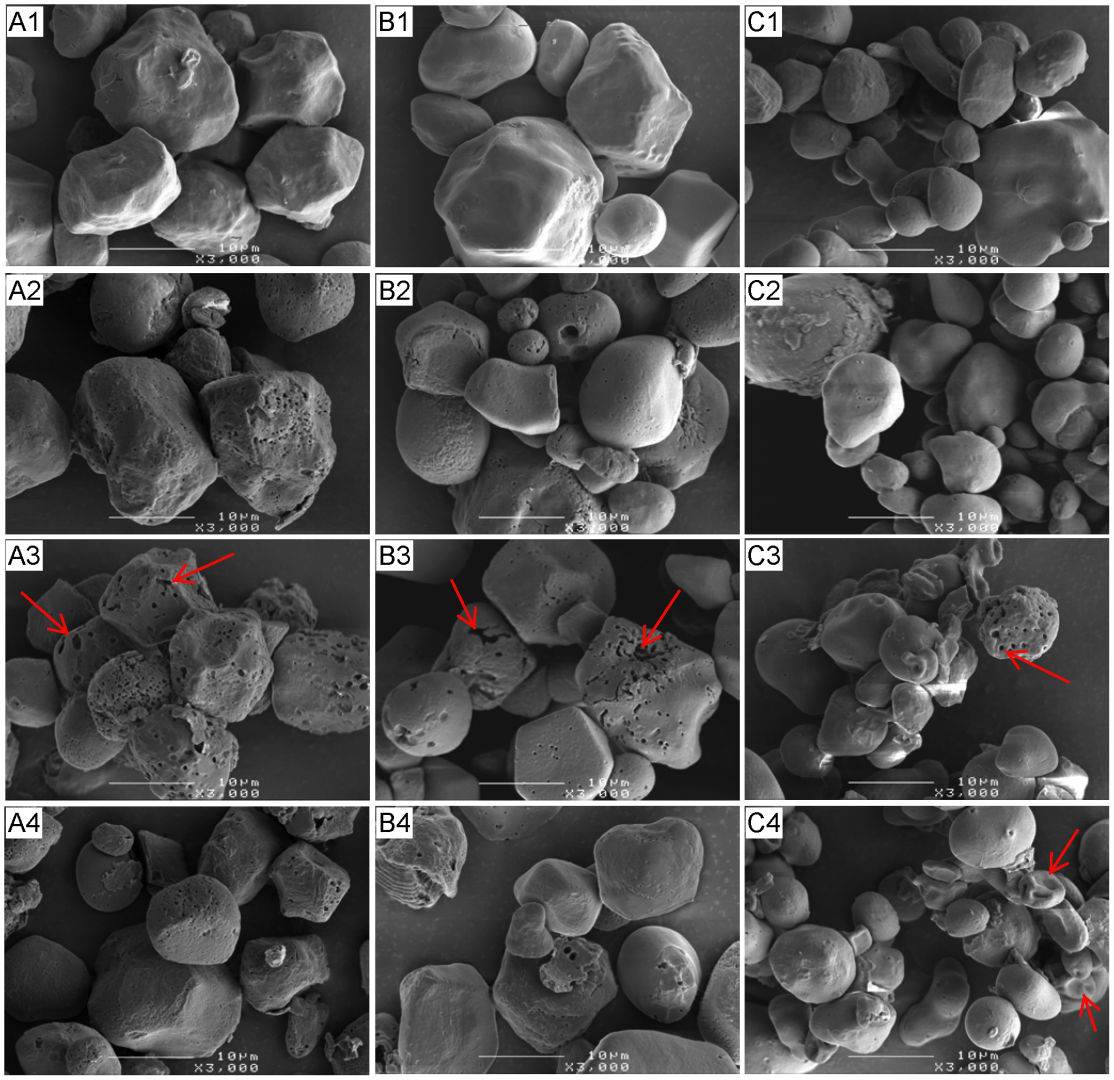
Scanning electron micrographs of starches partially hydrolyzed by pancreatic extract. Waxy (A), normal (B), and high-amylose maize (C) were incubated with pancreatic extract for 0 (native, A1, B1, C1), 2 (A2, B2, C2), 8 (A3, B3, C3), and 12 h (A4, B4, C4) at 37°C. Surface pores (arrows in A3, B3, and C3) were enlarged by the hydrolysis; there were more pores in waxy and normal maize starch. Surface erosion (arrow in C4) was observed in high-amylose maize.

**Figure 5:**
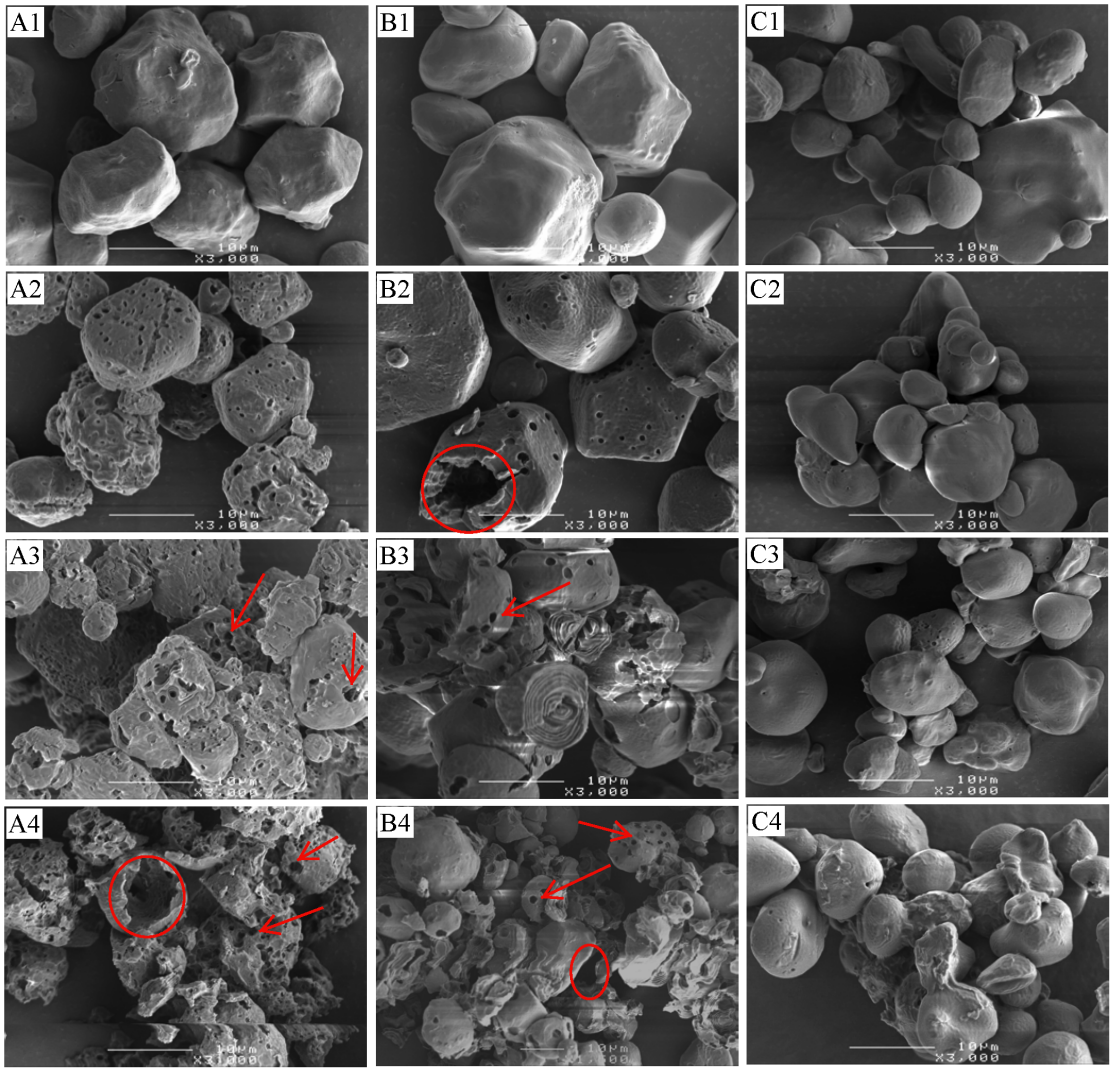
Scanning electron micrographs of starches partially digested by intestinal extract. Waxy (A), normal (B), and high-amylose maize (C) were incubated with intestinal extract for 0 (native, A1, B1, C1), 2 (A2, B2, C2), 8 (A3, B3, C3), and 12 h (A4, B4, C4) at 37°C. Surface pores (arrows in A3–4 and B3–4) of waxy and normal maize were much larger in comparison with Fig. 4; large cavities (circle) and fragments of these two maize starches were observed. More amounts of pores of high-amylsoe maize were seen in comparison with Fig. 4.

From SEM examinations, waxy maize and normal maize were regular in shape (mostly spherical and polygonal) with numerous pores on the surface (Fig. 4, A1 and B1). High-amylose maize (Fig. 4, C1), on the other hand, was observed to be irregular in shape without visible surface pores. After hydrolysis either by pancreatic or intestinal extracts, waxy maize and normal maize had clearly visible multiple holes (Fig. 4, A2 and B2; Fig. 5, A2 and B2) along the whole of the granule surface, whereas surface erosion and selective pitting were observed in high-amylose maize (Fig. 4, C2–4; Fig. 5, C2–4).

Pancreatic extract-treated starches were less extensively eroded (Fig. 4) consistent with the lower extent of starch hydrolysis (Fig. 2). Intestinal extract-treated waxy and normal maize had enlarged pores which merged to form larger holes (Fig. 5, A3 and B3) and finally to hollow granule remnants. Some of these internally corroded granules did not retain their granular structure on further digestion and were fragmented to irregularly shaped residues (Fig. 5, A4 and B4). High-amylose maize showed a similar digestion pattern with both pancreatic and intestinal extracts, involving surface roughening on initial hydrolysis followed by selective pitting of granules in the later phase of hydrolysis. Numerous granules with no obvious damage were observed alongside some disrupted granular fragments even after 12 h of hydrolysis (Fig. 4, C4; Fig. 5, C4).

### Molecular Size Distributions

**Figure 6:**
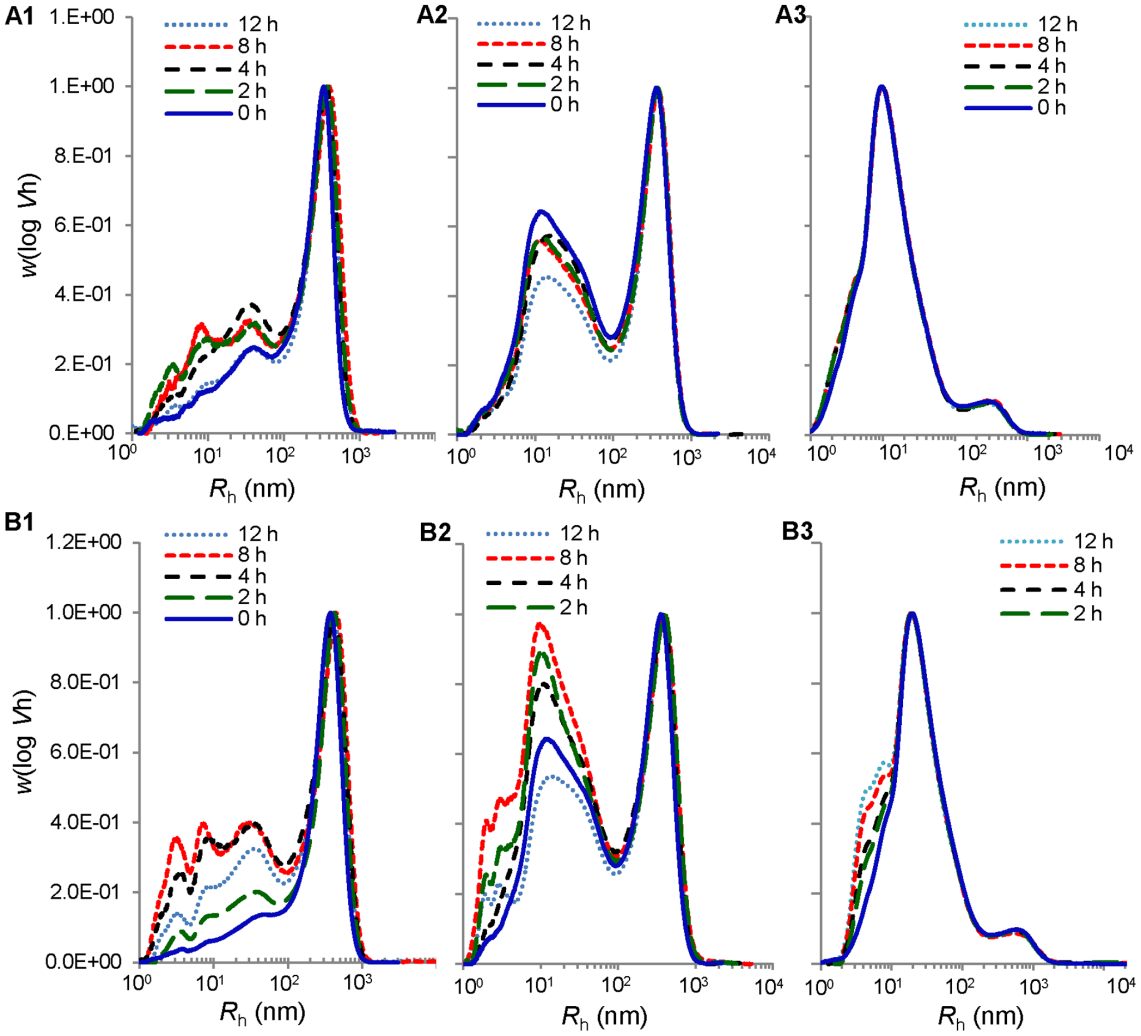
Molecular size distribution of un-debranched starch molecules. Waxy, normal, and high-amylose maize were incubated with pancreatic (A1, A2, and A3) and intestinal extracts (B1, B2, and g007B3) respectively for 0 (native), 2, 4, 8, and 12 h at 37°C.

**Figure 7:**
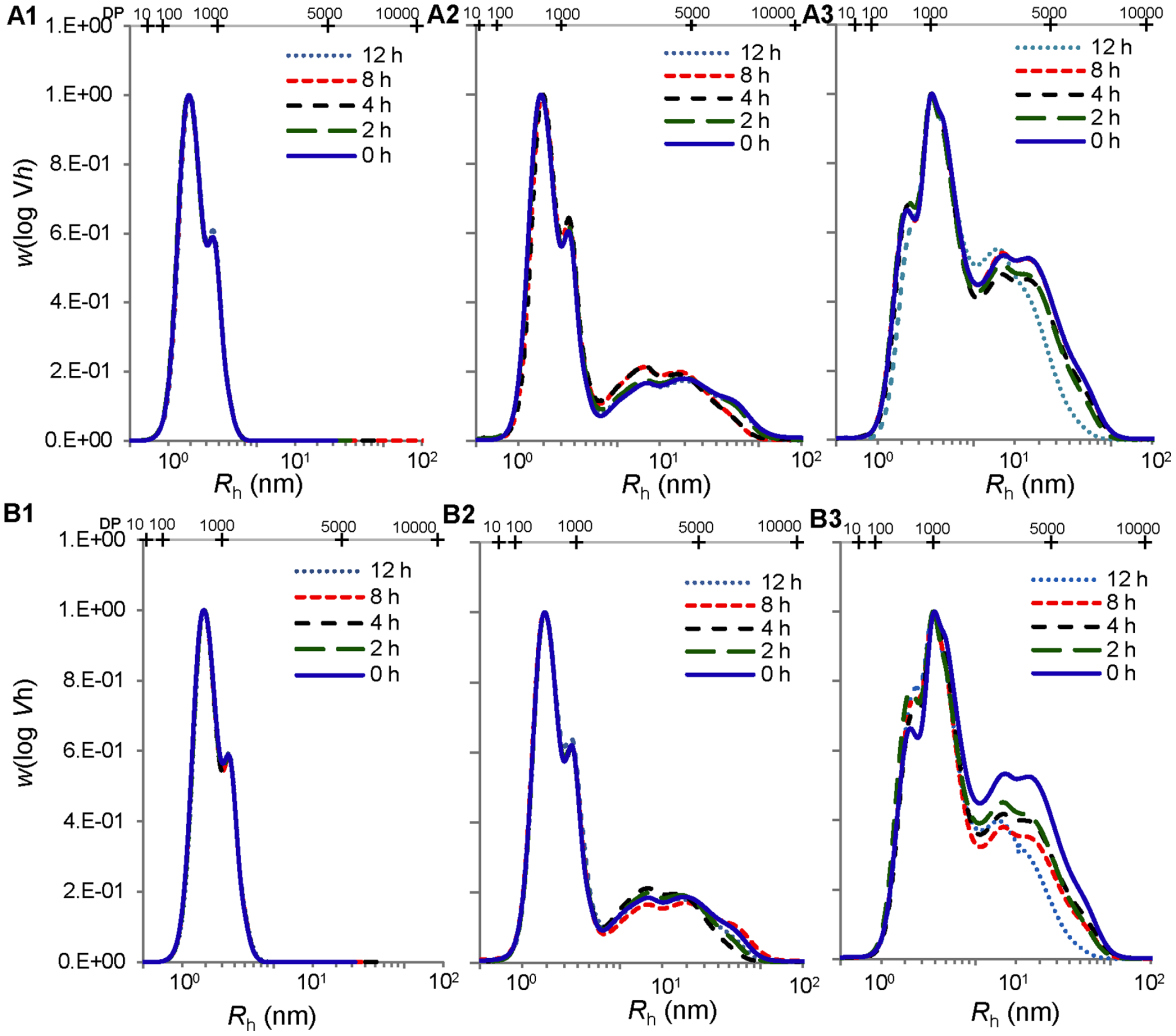
Molecular size distribution of debranched starch molecules. Waxy, normal, and high-amylose maize were incubated with pancreatic (A1, A2, and A3) and intestinal extracts (B1, B2, and B3) respectively for 0 (native), 2, 4, 8, and 12 h at 37°C, and then the residues were debranched by isoamylase. The chromatographs were also presented in terms of degree of polymerizations (DP) as the upper X-axis.

Molecular size distributions of un-debranched and debranched molecules in native starch and degraded starch remnants are shown in Figs. 6 and 7. The un-debranched molecular distribution of waxy maize, normal maize and high-amylose maize before and after digestion with either pancreatic or intestinal extract remained similar with two distinct peaks for amylose and amylopectin separated at ca. *R*_h_ = 200 nm. Both pancreatic and intestinal extract-treated waxy maize samples (Fig. 6, A1 and B1) showed an increase in the amount of intermediate-sized fraction, resulting from degradation of amylopectin molecules. The size of the largest intermediate-sized fraction (Figs. 6, A1 and B1) was about 9 nm consistent with the expected size of an amylopectin cluster, and was degraded at longer digestion time (12 h). Pancreatic and intestinal extract-treated normal maize samples showed a similar increase in intermediate-sized fractions (Figs. 6, A2 and B2), whereas the amylopectin cluster-sized fragments were not observed due to overlap in size with the amylose fraction. High-amylose maize did not have notable changes in size distribution, but the intestinal extract-treated chromatogram had a slight increase in smaller macromolecules of *R*_h_ less than 7 nm (Fig. 6, B3).

The debranched molecule distributions showed similar patterns during the hydrolysis period for both pancreatic and intestinal extract-treated waxy and normal maize (Fig. 7). Intestinal extract-treated high-amylose maize had a decrease at the *R*_h_ range 6–13 nm during the hydrolysis period (Fig. 7, B3).

### X-ray Diffraction Patterns

**Figure 8:**
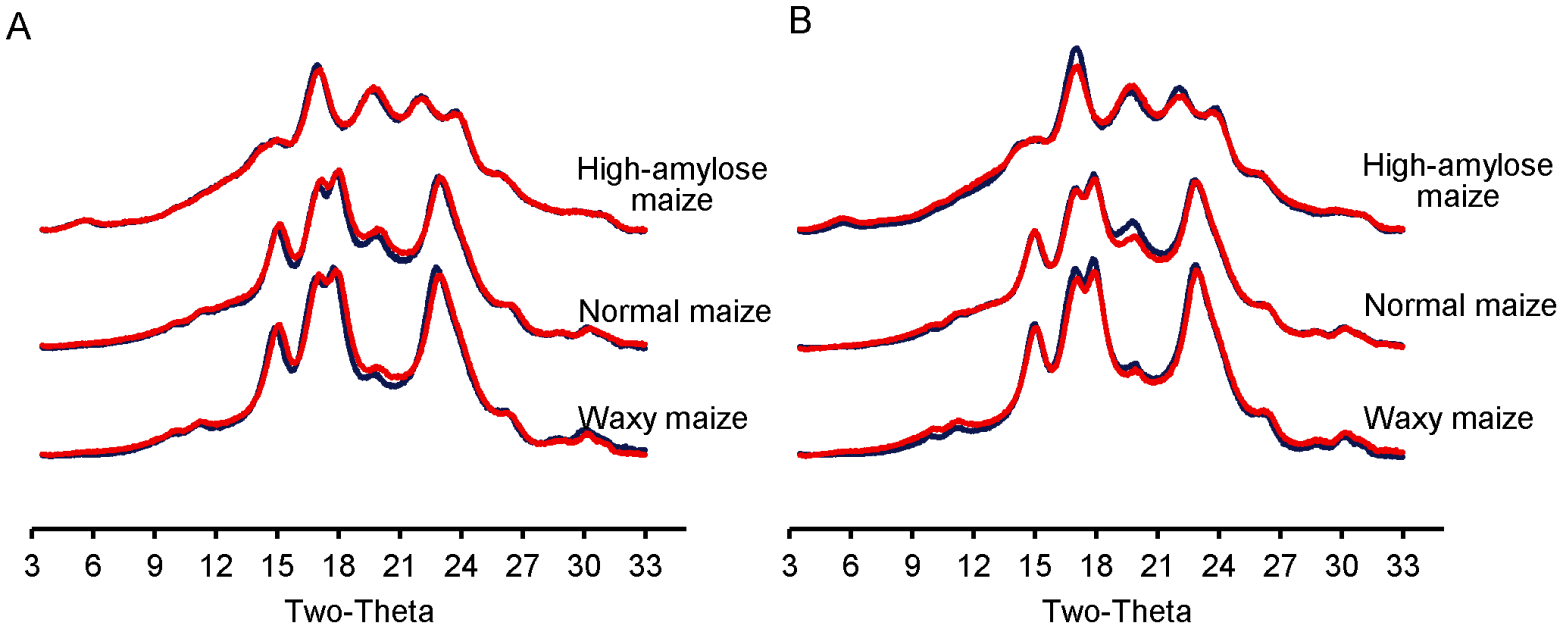
X-ray diffractograms of native and partially digested starch. Waxy, normal, and high-amylose maize were incubated with pancreatic (A) and intestinal extracts (B) respectively for 0 (native, red) and 12 h (blue) at 37°C.

Native waxy and normal maize starches exhibited the A-type crystalline diffraction pattern with a main diffraction doublet at 17 and 18° and peaks at 15, 20, and 23° 2θ (Fig. 8) [23]; native high-amylose maize starch had B-type crystalline pattern related to its longer amylopectin chains (compared to waxy maize and normal maize) with distinct peaks at ≈5, 17, 22 and 24° 2θ. Both native normal maize and high-amylose maize also had a detectable V-type diffraction peak at 20° 2θ, associated with ordered packing of amylose single helices [23].

Native waxy, normal and high-amylose maize starches had relative crystallinity values of 32.8, 23.8 and 11.9%, respectively, which increased slightly by 1.7, 0.5 and 3.5% after intestinal extract hydrolysis (12 h) (Table 2). For waxy maize, the increase in crystallinity was mainly contributed by an increase in peak area of the doublet at 17 and 18° 2θ; for normal maize, the increase in sharpness and peak area at 20° 2θ contributed to the increase in crystallinity (Fig. 8). This was primarily due to an increase in V-type crystallinity from 0.8 to 1.2% (Table 2). High amylose maize displayed a more intense diffraction peak at 17° and 22° after digestion, while the rest of the diffractogram remained largely unchanged (Fig. 8). The crystalline pattern (Fig. 8A), relative crystallinity and area contributed by major diffraction peaks of pancreatic extract-treated starch were very similar to native starch (data not shown).

**Table 2:** Relative crystallinity of starches partially digested by pancreatic and intestinal extracts.

Starch	Major diffraction peaks (degrees2θ)^1^							Minor peaks	Total Crystallinity
	5	15	17/18	20	22	23	24		
***Native Starch***									
Waxy maize	–	3.4	11.8	0.3	–	14.9	–	2.4	32.8
Normal maize	–	2.7	8.0	0.8	–	10.5	–	1.8	23.8
High-amylose maize	0.5	1.0	4.3	2.5	1.1	–	1.2	1.3	11.9
***Intestinal Extract Digestion***									
Waxy maize	–	3.7	12.7	0.3	–	15.2	–	2.6	34.5
Normal maize	–	2.7	8.1	1.2	–	10.5	–	1.8	24.3
High-amylose maize	0.4	1.3	6	2.5	1.8	–	1.6	1.8	15.4

1 Pancreatic extract digested starches and native starch had similar relatively crystallinity and area contributed by major diffraction peaks.

### Thermal Properties

**Figure 9:**
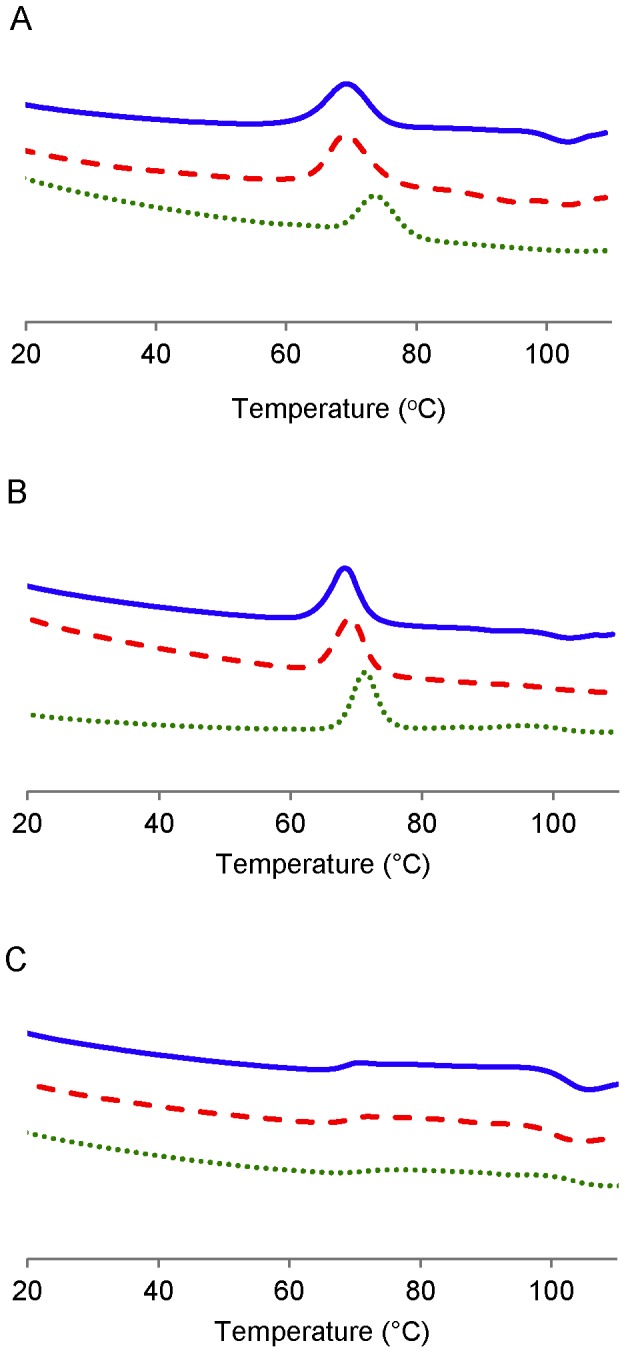
DSC endotherms of native and partially digested starch. Waxy, normal, and high-amylose maize were incubated with pancreatic (red, broken line) and intestinal extracts (green, dash line) respectively for 12 h at 37°C. The native starch is shown as blue solid line.

The DSC endotherms of granular starches before and after pancreatic and intestinal extract digestion for 12 h are shown in Fig. 9 and the parameters are tabulated in Table 3. The waxy and normal maize starches had a symmetric endotherm, whereas high-amylose maize starch had an asymmetric and broad thermogram (data is not shown in Table 3 due to uncertainty associated with characterization of enthalpy change and gelatinization temperature). The peak gelatinization temperatures of intestinal extract-treated waxy and normal maize starches increased by about 5°C and 3°C, respectively, and the peaks were slightly narrower (ca 1°C), with a slight decrease in ΔH observed for both starches (Table 3). In contrast, there were minimal changes in gelatinization temperature and enthalpy change in pancreatic extract-treated starches.

**Table 3:** Gelatinization parameters of starches.

	Native starch	Pancreatic extract digestion^1^	Intestinal extract digestion^1^
***Waxy maize***			
ΔH	11.78±0.012,b	13.07±0.36^a^	9.07±0.05^c^
To	63.01±0.03^c^	64.10±0.01^b^	68.71±0.02^a^
Tp	69.08±0.11^b^	68.93±0.01^b^	73.60±0.04^a^
Te	74.84±0.32^b^	75.62±0.02^b^	79.05±0.08^a^
ΔT^3^	11.83±0.29^a^	11.52±0.01^a^	10.35±0.11^b^
***Normal maize***			
ΔH	10.59±0.11^a^	10.89±0.21^a^	9.43±0.64^a^
To	63.92±0.20^c^	64.59±0.05^b^	67.67±0.09^a^
Tp	68.33±0.23^c^	68.94±0.10^b^	71.16±0.02^a^
Te	72.04±0.35^b^	72.90±0.07^b^	74.72±0.11^a^
ΔT	8.12±0.16^a^	8.32±0.12^a^	7.06±0.02^b^

1 12 h digestion.

2 Values are mean ± standard deviation of triplicated measurements; means do not share the same letter in each row were significantly different. The statistical assays were achieved using one-way ANOVA followed by the Tukey’s test with a significant level of 5%.

3 ΔT = Te – To.

## Discussion

This study supported the view [7]–[9], [24] that the mucosal α-glucosidases do not only passively convert post-α-amylase dextrin to glucose; they can act synergistically with α-amylase to digest granular starch at the initial hydrolysis stage although mucosal α-glucosidases, by itself, hydrolyzed only a few percentages of granular starch. This was exemplified in Fig. 1, where all four individual mucosal α-glucosidases were to some degree digest granular starch to free glucose in the absence of α-amylase. In addition, pancreatic- and intestinal-extract hydrolysis of the starches produced maltooligosaccharides of quite different kinetic patterns (Fig. 1, A3 and B3). Production of maltooligosaccharides in this experiment signified direct digestion of granular starch as opposed to glucose generation which is specific to the further action of the α-glucosidases. Pancreatic-extract hydrolysis approached an activity limitation (a plateau in Fig. 2, A3) in about 2 h, and the maltooligosaccharides amount of three maize starches were in the order of waxy maize>normal maize>high-amylose maize. On the other hand, intestinal extract digestion had a different kinetic pattern, which did not reach a plateau but showed further generation of maltooligosaccharides until 4 (high-amylose maize) and 8 h (waxy maize and normal maize) with a subsequent drop of released maltooligosaccharide amount (Fig. 2, B3). Thus, the presence of the α-glucosidases in the intestinal-extract, along with α-amylase, substantially increased the direct digestion of the granular starch.

Several possible mechanisms were proposed to explain the higher amount of maltooligosaccharide with intestinal extract in comparison with α-amylase. α-Glucosidase digestion might provide α-amylase a new route to bypass a granular structural barrier. From our previous study using the recombinant Nt-MGAM, a unique digestion pattern, different from α-amylase, was observed on granular starch [10]. Thus, it might create alternative routes for α-amylase hydrolysis of the starch granule. This might be connected with the observed increase in granule crystallinity and melting temperatures after intestinal extract hydrolysis, but not pancreatic extract hydrolysis. If mucosal α-glucosidases acted at different locations to α-amylase, this might allow some annealing of the starch structure, thereby causing an increase in the relative amount (Fig. 8) and thermal stability (Fig. 9; Table 3) of crystallites within the partially-digested granule. Morphology resulted in Figs. 4 and 5 supported the hydrolysis kinetics data above. Native granular waxy maize and normal maize had numerous pores on the surface, which facilitated the entry of enzymes inside the granules [25], [26]; while high-amylose maize granules did not have such visible surface pores. Thus, it was logical that waxy maize and normal maize had higher susceptibility to both pancreatic and intestinal extracts. Progression of hydrolysis in waxy maize and normal maize with intestinal extract resulted first in enlarged pores, then hollow granules remnants, and finally fragments of irregular residues. Waxy maize had relatively higher fragmentation than normal maize. The amylose in normal maize, which has higher distribution near the granule surface [27], showed low susceptibility to enzymes. Pancreatic extract-hydrolyzed waxy and normal maize had enlarged surface pores as well, but most granules were intact after the hydrolysis period (12 h). This agreed with maltooligosaccharide results that pancreatic hydrolysis approached a limitation at the initial period (2 h). We investigated the changes in starch molecular organization that occurred during digestions, including molecular size distribution and X-ray diffraction pattern of starch residues and thermal properties. As described above, crystallinity was increased slightly after hydrolysis with intestinal extract, consistent with independent action of α-amylase and mucosal α-glucosidases on granules. The change of molecular size of debranched and un-debranched molecules after hydrolysis showed patterns expected for α-amylase degradation as exo-acting α-glucosidase action had much less effect on overall molecular size than endo-acting α-amylase.

The mutually dependent and complementary relationship of α-amylase and mucosal α-glucosidase on granular starch digestion was proposed long ago by Brown *et al.* in 1879 [28]. α-Amylase rapidly breaks down starch molecules to maltose and other dextrins, which are very slowly and partially converted into glucose by a continuance of the mucosal α-glucosidase reaction. Here we showed that mucosal α-glucosidases also directly and slowly hydrolyzed starch to glucose. The intestinal extract hydrolysis produced glucose and maltooligosaccharides from three starches in different orders; the glucogenesis followed the order of normal maize>waxy maize>high-amylose maize; the maltooligosaccharides production followed the order of waxy maize>normal maize>high-amylose maize. The glucogenesis by the intestinal extract hydrolysis was a combination of direct hydrolysis of starch and the conversion of malto-oligosaccharides to glucose. These two types of hydrolytic reactions on various starches were different due to the difference of starch internal structures [29]. Taken together, this study showed that mucosal α-glucosidases are able to individually break down granular starch to some degree, and can alter the way in which α-amylase hydrolyzes granular starch and dextrins.

### Conclusions

Mucosal α-glucosidases were shown to not only passively convert post-α-amylase dextrins to glucose, but also to be able to participate in the initial stage of digestion of granular starch. It is known that the digestion of the post-α-amylase dextrins prevents the build-up of high concentrations of products that may inhibit α-amylolysis, and, thus α-amylase can keep acting until it reaches the structural barriers of starch such as branch points. However, recombinant mucosal α-glucosidases could also directly digest granular starch in the absence of α-amylase. This suggested that the mucosal α-glucosidases may have a larger complimentary role beyond that of simply generating glucose by assisting luminal α–amylase hydrolysis. The rate and extent of granular starch digestibility, then, likely depends on the presence and relative amounts of both α-amylase and mucosal α-glucosidases.

## Supporting Information

Table S1 **Kinetics of pancreatic and intestinal hydrolysis in releasing maltooligosaccharides from three granular starches.**
(PDF)

Table S2 **Kinetics of releasing maltooligosaccharides from three granular starches by pancreatic and intestinal extracts.**
(PDF)

Table S3 **Kinetics of pancreatic and intestinal hydrolysis in releasing maltose, maltotriose, and maltotetraose from three granular starches.**
(PDF)
