# Supplementary material for: Mammalian Mucosal α-Glucosidases Coordinate with α-Amylase in the Initial Starch Hydrolysis Stage to Have a Role in Starch Digestion beyond Glucogenesis
Source: PLoS One. 2013 Apr 25;8(4):e62546. doi: 10.1371/journal.pone.0062546 (PMC3636141; doi:10.1371/journal.pone.0062546)
Supplement: Table S1 — Kinetics of pancreatic and intestinal hydrolysis in releasing maltooligosaccharides from three granular starches. (PDF) [file pone.0062546.s001.pdf]

Table S1. Kinetics of pancreatic and intestinal hydrolysis in releasing maltooligosaccharides from three granular starches

	Pancreatic extract	Intestinal extract
Waxy maize		
2 h	156.34 ± 3.80[a]	176.65 ± 11.35[a]
4 h	161.68 ± 8.94[b]	293.57 ± 6.16[a]
8 h	155.71 ± 1.88[b]	332.84 ± 16.63[a]
12 h	161.74 ± 5.03[b]	207.74 ± 32.13[a]
Normal maize		
2 h	105.04 ± 3.92[b]	157.54 ± 10.09[a]
4 h	125.30 ± 10.03[b]	201.12 ± 16.55[a]
8 h	129.40 ± 4.77[b]	184.79 ± 12.69[a]
12 h	124.53 ± 2.83[a]	100.92 ± 19.00[a]
High-amylose maize		
2 h	39.46 ± 1.55[b]	90.59 ± 5.02[a]
4 h	58.26 ± 3.70[b]	119.29 ± 2.28[a]
8 h	64.77 ± 1.35[a]	34.37 ± 14.42[b]
12 h	62.21 ± 4.56[a]	0.00 ± 0.00[b]

Numbers are mean ± standard deviation of triplicated measurements. The statistical assays were achieved using one-way ANOVA followed by Tukey's test with a significant level of 5%. Means do not share the same letter in each row were significantly different.
