# Supplementary material for: Mammalian Mucosal α-Glucosidases Coordinate with α-Amylase in the Initial Starch Hydrolysis Stage to Have a Role in Starch Digestion beyond Glucogenesis
Source: PLoS One. 2013 Apr 25;8(4):e62546. doi: 10.1371/journal.pone.0062546 (PMC3636141; doi:10.1371/journal.pone.0062546)
Supplement: Table S2 — Kinetics of releasing maltooligosaccharides from three granular starches by pancreatic and intestinal extracts. (PDF) [file pone.0062546.s002.pdf]

Table S2. Kinetics of releasing maltooligosaccharides from three granular starches by pancreatic and intestinal extracts

	Waxy maize	Normal maize	High-amylose maize
Pancreatic extract			
2 h	156.34 ± 3.80[a]	105.04 ± 3.92[a]	39.46 ± 1.55[c]
4 h	161.68 ± 8.94[a]	125.30 ± 10.03[b]	58.26 ± 3.70[c]
8 h	155.71 ± 1.88[a]	129.40 ± 4.77[b]	64.77 ± 1.35[c]
12 h	161.74 ± 5.03[a]	124.53 ± 2.83[b]	62.21 ± 4.56[c]
Intestinal extract			
2 h	176.65 ± 11.35[a]	157.54 ± 10.09[b]	90.59 ± 5.02[c]
4 h	293.57 ± 6.16[a]	201.12 ± 16.55[b]	119.29 ± 2.28[c]
8 h	332.84 ± 16.63[a]	184.79 ± 12.69[b]	34.37 ± 14.42[c]
12 h	207.74 ± 32.13[a]	100.92 ± 19.00[b]	0.00 ± 0.00[c]

Numbers are mean ± standard deviation of triplicated measurements. The statistical assays were achieved using one-way ANOVA followed by Tukey's test with a significant level of 5%. Means do not share the same letter in each row were significantly different.
