# Supplementary material for: Mammalian Mucosal α-Glucosidases Coordinate with α-Amylase in the Initial Starch Hydrolysis Stage to Have a Role in Starch Digestion beyond Glucogenesis
Source: PLoS One. 2013 Apr 25;8(4):e62546. doi: 10.1371/journal.pone.0062546 (PMC3636141; doi:10.1371/journal.pone.0062546)
Supplement: Table S3 — Kinetics of pancreatic and intestinal hydrolysis in releasing maltose, maltotriose, and maltotetraose from three granular starches. (PDF) [file pone.0062546.s003.pdf]

Table S3. Kinetics of pancreatic and intestinal hydrolysis in releasing maltose, maltotriose, and maltotetraose from three granular starches

	0 h	2 h	4 h	8 h	12 h
**Pancreatic extract**					
Waxy maize					
maltose	0.002 ± 0.000^{b}	0.132 ± 0.031^{b}	0.43 ± 0.037^{a}	0.414 ± 0.010^{a}	0.490 ± 0.097^{a}
maltotriose	0.003 ± 0.000^{b}	0.110 ± 0.022^{b}	0.332 ± 0.039^{a}	0.306 ± 0.025^{a}	0.355 ± 0.070^{a}
maltotetraose	0.002 ± 0.000^{c}	0.044 ± 0.004^{b}	0.086 ± 0.008^{a}	0.090 ± 0.010^{a}	0.110 ± 0.012^{a}
Normal maize					
maltose	0.002 ± 0.001^{b}	0.129 ± 0.031^{b}	0.484 ± 0.154^{a}	0.451 ± 0.064^{a}	0.493 ± 0.054^{a}
maltotriose	0.001 ± 0.001^{c}	0.096 ± 0.020^{bc}	0.337 ± 0.118^{ab}	0.345 ± 0.053^{a}	0.349 ± 0.037^{a}
maltotetraose	0.001 ± 0.000^{b}	0.031 ± 0.003^{ab}	0.116 ± 0.023^{a}	0.132 ± 0.055^{a}	0.108 ± 0.024^{ab}
High-amylose maize					
maltose	0.001 ± 0.000^{b}	0.112 ± 0.026^{ab}	0.223 ± 0.113^{ab}	0.330 ± 0.109^{a}	0.326 ± 0.076^{a}
maltotriose	0.001 ± 0.001^{a}	0.073 ± 0.014^{a}	0.159 ± 0.022^{a}	0.216 ± 0.134^{a}	0.142 ± 0.028^{a}
maltotetraose	non-detected	0.007 ± 0.000^{a}	0.022 ± 0.002^{a}	0.056 ± 0.049^{a}	0.021 ± 0.000^{a}
**Intestinal extract**					
Waxy maize					
maltose	0.002 ± 0.001^{b}	0.184 ± 0.032^{b}	0.816 ± 0.099^{a}	0.999 ± 0.145^{a}	1.159 ± 0.086^{a}
maltotriose	0.003 ± 0.001^{b}	0.080 ± 0.004^{b}	0.335 ± 0.018^{a}	0.317 ± 0.070^{a}	0.356 ± 0.062^{a}
maltotetraose	0.002 ± 0.000^{a}	0.043 ± 0.004^{a}	0.066 ± 0.004^{a}	0.030 ± 0.043^{a}	non-detected
Normal maize					
maltose	0.002 ± 0.001^{c}	0.175 ± 0.032^{bc}	0.571 ± 0.043^{ab}	0.950 ± 0.167^{a}	1.019 ± 0.231^{a}
maltotriose	0.001 ± 0.000^{b}	0.086 ± 0.018^{b}	0.228 ± 0.011^{a}	0.279 ± 0.058^{a}	0.255 ± 0.046^{a}
maltotetraose	0.001 ± 0.000^{c}	0.032 ± 0.003^{b}	0.040 ± 0.005^{ab}	0.047 ± 0.002^{a}	non-detected
High-amylose maize					
maltose	0.001 ± 0.000^{b}	0.102 ± 0.028^{ab}	0.224 ± 0.065^{a}	0.173 ± 0.004^{a}	0.215 ± 0.007^{a}
maltotriose	0.001 ± 0.000^{b}	0.020 ± 0.002^{b}	0.071 ± 0.018^{a}	0.053 ± 0.001^{a}	non-detected
maltotetraose	non-detected	non-detected	non-detected	non-detected	non-detected

Numbers are mean ± standard deviation of triplicated measurements. The statistical assays were achieved using one-way ANOVA followed by Tukey's test with a significant level of 5%. Means do not share the same letter in each row were significantly different.
